# Thrombus migration in ischemic stroke due to large vessel occlusion: a question of time

**DOI:** 10.1136/jnis-2022-019365

**Published:** 2022-11-01

**Authors:** Christoph Riegler, Eberhard Siebert, Justus F Kleine, Christian H Nolte

**Affiliations:** 1 Klinik und Hochschulambulanz für Neurologie, Charite Universitatsmedizin Berlin, Berlin, Germany; 2 Center for Stroke Research Berlin (CSB), Charite Universitatsmedizin Berlin, Berlin, Germany; 3 Institut für Neuroradiologie, Charite Universitatsmedizin Berlin, Berlin, Germany

**Keywords:** Thrombectomy, Thrombolysis, Stroke, Intervention

## Abstract

**Background:**

Thrombus migration (TM) is frequently observed in large vessel occlusion (LVO) ischemic stroke to be treated by endovascular thrombectomy (EVT). TM may impede complete recanalization and hereby worsen clinical outcomes. This study aimed to delineate factors associated with TM and clarify its impact on technical and functional outcome.

**Methods:**

All patients undergoing EVT due to LVO in the anterior circulation at two tertiary stroke centers between October 2015 and December 2020 were included. Source imaging data of all individuals were assessed regarding occurrence of TM by raters blinded to clinical data. Patient data were gathered as part of the German Stroke Registry, a multicenter, prospective registry assessing real-world outcomes. Technical outcome was assessed by modified Thrombolysis in Cerebral Infarction scale (mTICI). Functional outcome was assessed by modified Rankin Scale (mRS) at 3 months.

**Results:**

The study consisted of 512 individuals, of which 71 (13.8%) displayed TM. In adjusted analyses, TM was associated with longer time from primary imaging to reassessment in the angio suite (aOR 2.37 (1.47 to 3.84) per logarithmic step) and intravenous thrombolysis (IVT; aOR 4.07 (2.17 to 7.65)). In individuals with IVT, a needle-to-groin time >1 hour was associated with higher odds for TM (aOR 2.60 (1.20 to 5.99)). TM was associated with lack of complete recanalization (aOR_mTICI3_ 0.46 (0.24 to 0.90)) but TM did not worsen odds for good clinical outcome (aOR_mRS≤2_d90_ 0.89 (0.47 to 1.68)).

**Conclusions:**

TM is associated with IVT and longer time between sequential assessments of thrombus location. Consequently, TM may be of high relevance in patients with drip-and-ship treatment.

**WHAT IS ALREADY KNOWN ON THIS TOPIC**

- Thrombus migration (TM) is associated with intravenous thrombolysis and seems to be beneficial for clinical outcomes despite lower rates of complete recanalization.

**WHAT THIS STUDY ADDS**

- TM is a time-dependent phenomenon and occurs particularly in patients with longer delay between imaging/thrombolysis and groin puncture.

**HOW THIS STUDY MIGHT AFFECT RESEARCH, PRACTICE OR POLICY**

- TM may be of high relevance in patients with drip-and-ship regime and could affect the impact of bridging therapy in these patients.

## Introduction

Due to positive evidence in several randomized controlled trials (RCTs), endovascular therapy (EVT) has become an essential part of acute management in ischemic stroke.^1^ Evidence shows that complete recanalization (modified Thrombolysis in Cerebral Infarction scale (eTICI) 3) after EVT is superior to successful, but incomplete, recanalization (mTICI 2b/2c) with regard to clinical outcomes.^2^ Recently, a phenomenon termed thrombus migration (TM), that is, downstream movement of the occluding thrombus in between initial imaging (computed tomography/magnetic resonance imaging-based angiography (CTA/MRA)) and pretreatment imaging in the angio suite, has attracted interest. TM in large vessel occlusion (LVO) stroke undergoing EVT was associated with reduced likelihood to achieve complete recanalization (mTICI 3) and intravenous thrombolysis (IVT) seemed to be the strongest factor facilitating TM.^3–8^ This has led to skepticism whether IVT should be applied before EVT^9 10^ as is recommended in current guidelines.^11^

In two prospective multicenter registry studies, TM was associated with a better clinical outcome at 3 months, despite significantly lower rates of complete recanalization.^3 4^ One of the abovementioned studies performed sequential CTA imaging of patients treated with IVT and observed an association between TM and a longer time from administration of IVT to subsequent imaging, suggesting time dependency of TM occurrence.^3^ Such an association with time could be relevant for stroke management concepts involving drip-and-ship or mobile stroke units. Consequently, the INTERRSECT investigators suggested that “thrombus dynamics over time should be further evaluated”.^3^

## Objective

This study aimed to assess prevalence and associated factors of TM as well as its impact on technical and functional outcome after EVT.

## Methods

### Study population and variables

Patients were identified from two centers of our local EVT registry, which is part of the German Stroke Registry (GSR-MT), a prospective, multicenter observational registry, that has been described in detail previously.^12^ The GSR-MT includes all individuals admitted to its participating centers with LVO, aged ≥18 years in whom EVT is initiated. A systematic follow-up regarding functional status 3 months after stroke via modified Rankin Scale (mRS) is regularly performed. All patients with sufficient imaging quality, and visible thrombus in both CTA/MRA as well as first series of digital subtraction angiography (DSA), were included into the current analysis. Stroke severity was assessed using the National Institutes of Health Stroke Scale (NIHSS). Functional independency was defined as mRS≤2 three months after stroke. As safety variables, we defined in-hospital death, death 3 months after stroke, any intracranial hemorrhage (ICH), and symptomatic ICH (sICH; ICH with worsening of NIHSS of four or more points or deterioration of vigilance due to a midline shift >2 mm caused by ICH).

### Imaging evaluation

In accordance with previous studies, any downstream movement of the most proximal visible thrombus was defined as TM.^3 4 6^ TM was categorized dichotomously following previously published data from the MR CLEAN Registry (TM vs no TM).^4^ Exact localization of the occluding thrombus was classified along different segments of the internal carotid artery (ICA) and middle cerebral artery (MCA) as detailed in the online supplemental file 1. A trained neurologist (CR) – blinded to clinical data and IVT status – reassessed all source imaging data regarding exact site of occlusion in CTA/MRA and DSA. Selected, more complicated, or ambiguous cases were reassessed by a senior neuroradiologist (JFK). Final recanalization status was classified according to mTICI score and categorized as unsuccessful (mTICI≤2a), successful (mTICI 2b/3), and complete recanalization (mTICI 3).

10.1136/jnis-2022-019365.supp1 Supplementary data

### Statistical analysis

Continuous baseline variables and treatment times are presented as median (IQR) and dichotomous variables as absolute numbers and percentage. Comparisons regarding distribution between groups were performed by Kruskal–Wallis and Chi-Quadrat test. Binary logistic regression analyses were carried out to assess the impact of TM on clinical and technical outcomes. Odds ratios (ORs) for clinical and safety outcomes were adjusted for age, sex, stroke severity (NIHSS at admission), IVT, and pre-event degree of dependency (mRS pre-stroke). For technical outcomes, adjustments were made for location of occlusion site (distal vs proximal), large artery atherosclerosis (LAA) as stroke etiology, successful recanalization after first pass, and time from groin to flow restoration. For addressing the impact of target occlusion on TM, occlusion site was dichotomized into proximal (ICA and MCA-M1-proximal) and distal (MCA-M1-distal/MCA-M2). Treatment times were transformed by natural logarithm whenever an approximate parametric distribution could be reached by this method. In subgroups with reduced numbers, the influence of treatment times was estimated by using cutoff values and tertiles. All analyses were carried out using IBM SPSS Statistics for Windows version 27.0. (IBM Corp., Armonk, NY).

### Informed consent and ethics approval

As stated elsewhere, the GSR-MT registry was centrally approved by the Ethics Committee of the Ludwig-Maximilians University LMU, Munich (689-15), as the leading ethics committee.^12^ Informed consent was not mandatory in accordance with local rules and regulations. Data sampling from patients undergoing EVT is mandated by federal law. Thus, selection bias through lack of informed consent could be minimized.^13^

## Results

**Figure 1:**
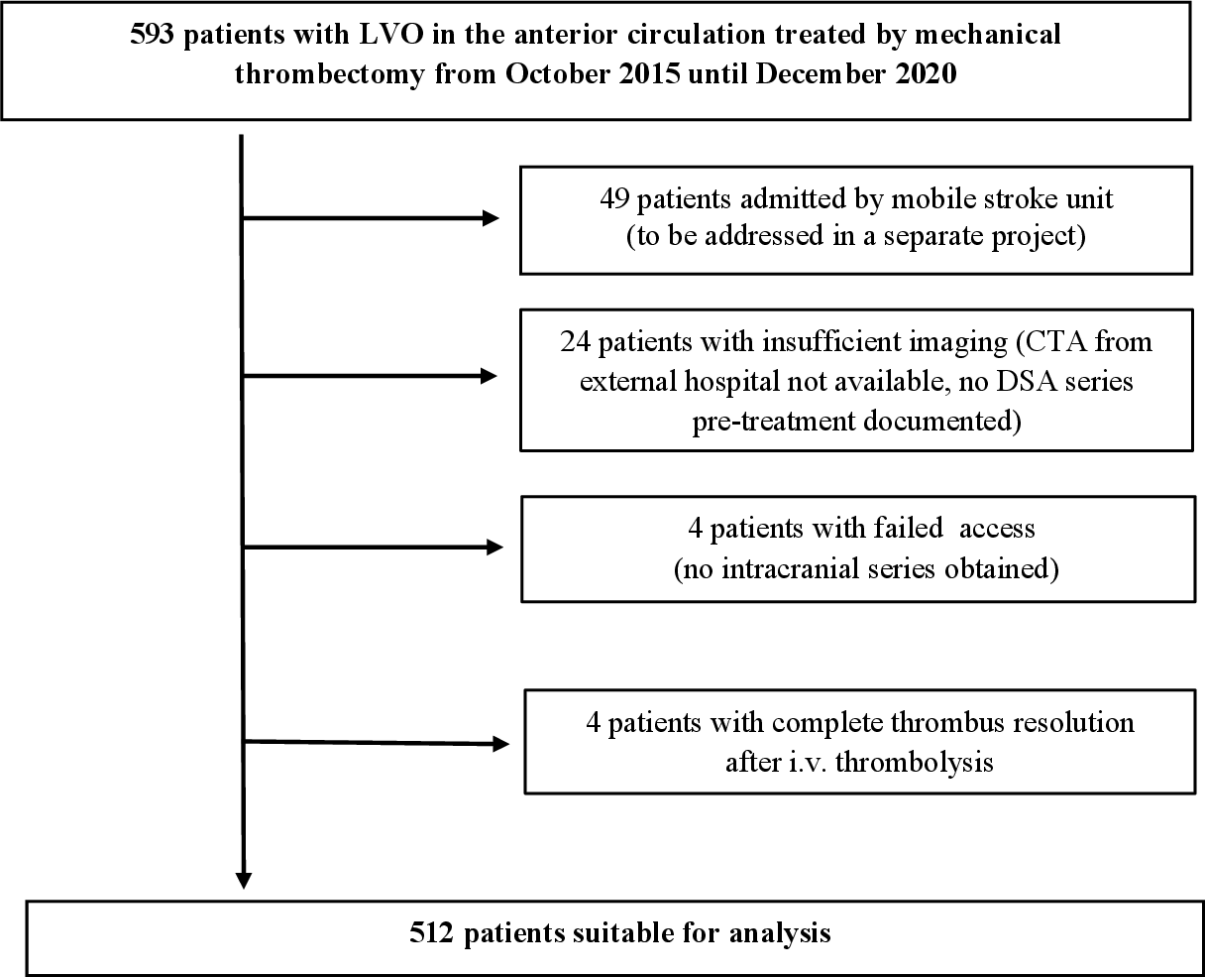
Selection of patients with large vessel occlusion (LVO) suitable for analysis. CTA, computed tomography angiography; DSA, digital subtraction angiography.

Between October 2015 and December 2020, 597 patients with LVO stroke in the anterior circulation received EVT at the two participating hospitals. Patients with insufficient imaging data, failed access to cerebral vessels, complete reperfusion in first series of DSA, or admission by a mobile stroke unit were excluded from the analysis. Figure 1 shows the selection of patients suitable for the final analysis. TM occurred in 71/512 (13.9%) patients. There was no significant difference between patients with or without TM regarding age, sex, baseline NIHSS, mRS pre-stroke, Alberta Stroke Program Early CT score (ASPECTS), and mode of admission (mothership vs drip-and-ship). Patients with TM had significantly higher rates of known onset of stroke (KOS; 70.4% vs 51.7%, P<0.01), IVT (74.6% vs 47.6%, P<0.01), and general anesthesia (78.3% vs 65.0%, P=0.03). All baseline variables as well as procedural details are depicted in table 1.

**Table 1:** Baseline variables, imaging, procedures, and outcomes

Parameter	No thrombus migration (n=441)	Thrombus migration (n=71)	P
Age (years), median (IQR)	**76** (66–83)	**75** (65–83)	0.88
Female sex, n (%)	250 (**56.7**)	34 (**47.9**)	0.17
Pre-stroke mRS≤2, n (%)	369 (**83.7**)	61 (**85.9**)	0.63
NIHSS at admission, median (IQR)	**15** (10–19)	**16** (10–20)	0.17
Arterial hypertension, n (%)	351 (**79.6**)	54 (**76.1**)	0.71
Diabetes mellitus, n (%)	94 (**21.3**)	11 (**15.5**)	0.48
Dyslipidemia, n (%)	213 (**48.3**)	34 (**47.9**)	0.92
Atrial fibrillation, n (%)	224 (**50.8**)	38 (**53.5**)	0.85
Current smoking, n (%)	61 (**14.5**)	5 (**7.6**)	0.20
LAA as stroke etiology, n (%)	149 (**33.8**)	17 (**23.9**)	0.10
Antiplatetelet agent, n (%)	129 (**29.3**)	21 (**29.6**)	0.95
Anticoagulation,* n (%)	88 (**20.0**)	8 (**11.3**)	0.08
Primary admission at interventional hospital, n (%)	399 (**90.5**)	63 (**88.7**)	0.65
MRI as primary imaging, n (%)	70 (**15.9**)	17 (**23.9**)	0.09
ASPECTS, median (IQR)	**8** (6-10)	**8** (6-9)	0.80
Known onset of stroke, n (%)	228 (**51.7**)	50 (**70.4**)	**<0.01**
Intravenous thrombolysis, n (%)	210 (**47.6**)	53 (**74.6**)	**<0.01**
Onset-to-needle time (min), median (IQR)	**90** (70–117)	**80** (66–105)	0.11
General anesthesia, n (%)	280/431 (**65.0**)	54/69 (**78.3**)	**0.03**
Use of stent-retrievers,† n (%)	292/429 (**68.1**)	42/56‡ (**75.0**)	0.29
Onset-to-groin time (min), median (IQR)	**175** (140–240)	**165** (132–216)	0.23
Imaging-to-groin time (min), median (IQR)	**83** (63–112)	**87** (72–121)	0.10
Needle-to-groin time (min), median (IQR)	**70** (48–95)	**75** (62–106)	0.10
Groin to flow restoration time (min), median (IQR)	**30** (20–54)	**36** (25–52)	0.51
Symptom onset to flow restoration time (min), median (IQR)	**208** (166–285)	**190** (154–259)	0.18

Clinical, technical, and safety outcomes	No thrombus migration (n=441)	Thrombus migration (n=71)	aOR (95% CI)	P
mRS≤2 at day 90, n (%)	133/428 (**31.1**)	22/69 (**31.9**)	0.89 (0.47 to 1.68)	0.72
Successful reperfusion (mTICI 2b/3), n (%)	375 (**86.0**)	62 (**91.2**)	3.35 (0.78 to 14.46)	0.11
Complete reperfusion (mTICI 3), n (%)	241 (**55.3**)	26 (**38.2**)	0.46 (0.24 to 0.90)	**0.02**
Death during hospital stay, n (%)	94 (**21.4**)	13 (**18.3**)	0.74 (0.36 to 1.55)	0.43
Death at day 90, n (%)	136/428 (**31.8**)	21/428 (**31.6**)	0.95 (0.49 to 1.83)	0.89
sICH, n (%)	24/441 (**5.4**)	5/71 (**7.0**)	0.99 (0.35 to 2.78)	0.98
Any ICH, n (%)	54/441 (**12.2**)	6/71 (**8.5**)	0.55 (0.22 to 1.37)	0.20
Parenchymal hematoma, n (%)	**37** (**8.4**)	**5 (7,0**)		
Subarachnoid hemorrhage, n (%)	**17** (**3.9**)	**1** (**1.4**)		

p-values in bold type denote statistical significance.

*Apixaban, dabigatran, edoxaban, rivaroxaban, phenprocoumon, heparin.

†Stent-retrievers vs aspiration only.

‡Mechanical thrombectomy not attempted in 15 patients (occlusion site too distal).

aOR, adjusted odds ratio; ASPECTS, Alberta Stroke Program Early CT score; CI, confidence interval; ICH, intracranial hemorrhage; IQR, interquartile range; LAA, large artery atherosclerosis; MRI, magnetic resonance imaging; mRS, modified Rankin Scale; mTICI, modified Thrombolysis in Cerebral Infarction; NIHSS, National Institutes of Health Stroke Scale; sICH, symptomatic intracranial hemorrhage.

The prevalence of TM was higher the further distal the initial target occlusion was located, except for ICA occlusions with patent circle of Willis. ICA occlusions with patent circle of Willis showed a relative high proportion of TM (extracranial ICA 13.6%, intracranial ICA with open carotid-T (ICA-I) 66.6%). Of note, in 7 of 11 patients (63.6%) with TM from the ICA into the circle of Willis, TM occurred relatively late (during stroke unit monitoring) as EVT had not been indicated initially, because patients were considered clinically stable. Only after clinical deterioration, imaging was repeated, TM was detected, and EVT performed. Details regarding initial target occlusions and TM patterns are listed in table 2. In 15/71 (21.1%) patients with TM, mechanical thrombectomy (MT) was not attempted, since thrombi were considered to be located too distally. In the remaining 56 patients with TM, mechanical treatment modality were stent-retrievers in 42 (75.0%) and thrombus aspiration only in 14 (25.0%) patients. In individuals without TM, thrombus aspiration as sole treatment was numerically more common (31.9%); however, the difference was not significant (P=0.29).

**Table 2:** Patterns and prevalence of thrombus migration stratified for segment*

Most proximal target occlusion	Digital subtraction angiography											
		ICA extracranial	ICA-I	ICA-T	Fetal PComA	MCA–M1 proximal	MCA–M1 distal	MCA–M2 proximal	MCA–M2 distal	M3/M4	Prevalence of thrombus migration (n (%))	
**CT-/MR-Angiography**	ICA extracranial	44				4	1	1			6 (**13.6**)	35 (**11.9**)
	ICA-I		10	3	1	1					5 (**66.6**)	
	ICA-T	-		94	-	8	2	–	–	–	10 (**9.6**)	
	MCA–M1 proximal	–		–	–	111	7	4	2	1	14 (**11.2**)	
	MCA–M1 distal	–		–	–	–	97	11	5	3	19 (**16.4**)	36 (**16.8**)
	MCA–M2 proximal	–		–	–	–	–	66	5	9	14 (**20.0**)	
	MCA–M2 distal	–		–	–	–	–	–	15	3	3 (**16.6**)	

*Three patients had an isolated anterior cerebral artery (ACA) occlusion, all without thromus migration.

CT/MRA, computed tomography/magnetic resonance imaging-based angiography; ICA, internal carotid artery; MCA, middle cerebral artery; PComA, posterior communicating artery.

**Figure 2:**
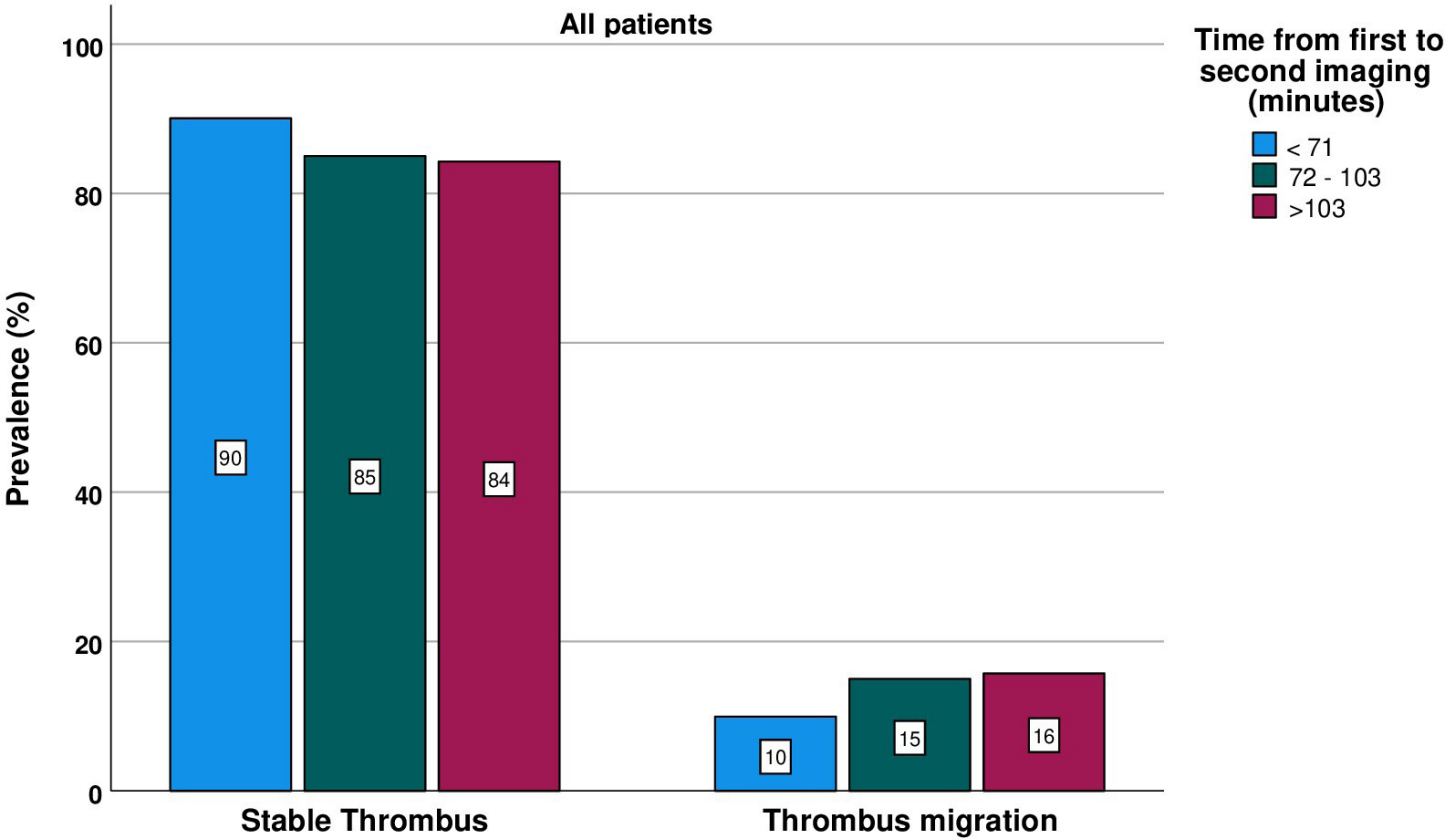
Time dependency of thrombus migration stratified by tertiles.

Factors associated with a higher rate of TM in multivariable analyses were IVT (aOR 4.07 (2.17 to 7.65), P<0.001) and longer time between first and subsequent imaging (aOR 2.37 (1.47 to 3.84) per logarithmic step, P<0.001). LAA stroke was associated with lower rates of TM (aOR 0.53 (0.28 to 1.00), P=0.05), borderline significant only. TM was associated with a more distal occlusion site in the subgroup of patients with IVT, but this association just failed to reach statistical significance in the total population (aOR 1.68 (0.96 to 2.95), P=0.07). In patients that had received IVT, needle-to-groin time had a stronger association with TM than imaging-to-groin time. While a clear linear effect of needle-to-groin time could not be found when analyzing all IVT-treated patients, we found a significant increase of TM-likelihood in patients with a needle-to-groin time of more than 1 hour (aOR 2.60 (1.20 to 5.99), P=0.02). When further restricting analysis to IVT-treated patients with KOS, a linear effect of needle-to-groin time on TM was observed (adjusted P for trend=0.03) (for further details see table 3). The occurrence of TM was time dependent in all patients, but the strongest association with time was seen in patients with KOS. Time dependency of TM is depicted in figure 2 for all patients and for subgroups with KOS and IVT in the online supplemental figure S1.

**Table 3:** Factors associated with thrombus migration

Parameter	All patients (n=478)		Patients with KOS (n=257)		Patients with IVT (n=232)		Patients with IVT and KOS (n=169)	
	aOR (95% CI)	P	aOR (95% CI)	P	aOR (95% CI)	P	aOR (95% CI)	P
IVT	4.07 (2.17 to 7.65)	**<0.001**	4.41 (1.74 to 11.15)	**<0.01**	–	–		
Ln‡ (time between sequential imaging)*	2.37 (1.47 to 3.84)	**<0.001**	2.36 (1.31 to 4.23)	**<0.001**	–	–		
Needle-to-groin time >60 min	–	–	–	–	2.60 (1.20 to 5.99)	**0.02**		
Needle-to-groin time (tertiles)					–	–	Adjusted P for trend	**0.03**
							Tertile 3 vs Tertile 1 4.03 (1.40 to 11.58)	**0.01**
							Tertile 2 vs Tertile 1 2.86 (0.98 to 8.34)	0.05
Distal location of LVO†	1.68 (0.96 to 2.95)	0.07	1.96 (0.99 to 3.89)	0.07	2.14 (1.10 to 4.15)	**0.03**	2.69 (1.23 to 5.85)	**0.01**
LAA etiology	0.53 (0.28 to 1.00)	0.051	0.50 (0.28 to 1.00)	0.053	0.54 (0.28 to 1.13)	0.10	0.45 (0.19 to 1.10)	0.08

Numbers in bold type denote statistical significance.

*Imaging-to-groin or imaging-to-imaging time.

†Distal segment of MCA M1 and MCA M2/3/4 segments vs ICA and proximal segment of MCA M1.

‡natural logarithm

.aOR, adjusted odds ratio; CI, confidence interval; ICA, internal carotid artery; IVT, intravenous thrombolysis; KOS, known onset of stroke; LAA, large artery atherosclerosis; LVO, large vessel occlusion; MCA, middle cerebral artery.

While TM patients showed numerically higher rates of successful reperfusion (aOR_mTICI≥2b_ 3.46 (0.81 to 14.78), P=0.09), rates of complete reperfusion were significantly lower in patients with TM (aOR_mTICI3_ 0.46 (0.24 to 0.90), P=0.02). In multivariable logistic regression analysis, TM was not associated with a relevant change in functional independency after 3 months (aOR _mRS ≤ 2_ 0.89 (0.47 to 1.68), P=0.72). In subgroups with either proximal or distal occlusion site we found no association of TM with good clinical outcome (aOR_mRS≤2_ 0.58 (0.21 to 1.59), P=0.29 (proximal occlusions) vs 0.94 (0.39 to 2.37), P=0.94 (distal occlusions)). Regarding the association of TM and complete recanalization (mTICI 3), subgroup analyses failed to reach significance (aOR_mTICI3_ 0.55 (0.24 to 1.26), P=0.16 (proximal occlusion) vs 0.34 (0.10 to 1.09), P=0.07 (distal occlusion)). Risk for death and both sICH and any ICH did not differ significantly between groups. We report exact numbers in table 1.

## Discussion

Our study had several findings: TM occurred in 1 of 7 stroke patients with LVO. Patients experiencing TM were four times more likely to have received IVT, had longer time intervals between first imaging and subsequent imaging, and seemed to be less likely to have LAA stroke etiology. Distal occlusion site was associated with TM in subgroups only. Clinical outcomes did not differ with respect to occurrence of TM, although rates of complete recanalization (mTICI 3) were significantly lower in the presence of TM.

Reports on the frequency of TM vary considerably in the current literature. Numbers range from 11% in an imaging study to 22% in the MR CLEAN Registry and even 54% in the INTERRSECT study.^3–5^ The rate of TM in our study is comparably low (14%). Variation may be explained by substantial differences regarding rate of IVT, mode of admission, and procedure times. Our rate of IVT (51%) was considerably lower than in the latter two of the abovementioned three studies (77% and 100%).^3 4^ The drip-and-ship rate was considerably higher (53.2%) in the MR CLEAN Registry than in our sample (9.8%), causing longer onset-to-groin times.^4^ Moreover, the INTERRSECT study had distinctly longer times from first imaging to reassessment (123 (61–236) min vs 84 (65–113) min in our study).^3^

### Factors associated with TM

In accordance with previous studies, IVT was the strongest predictor of TM in our sample.^3–5 14 15^ We found a higher TM rate in patients with longer time in between subsequent imaging and needle-to-groin puncture, respectively. While the impact of time on TM has been mentioned once in patients treated with IVT only,^3^ this is the first study extending the finding of time dependency to a mixed cohort of patients with and without IVT. As recently pointed out by Ciccone, all RCTs testing non-inferiority of MT without bridging therapy excluded patients transferred from external hospitals.^9 10 16–18^ However, in two large European registry studies, about half of the patients receiving MT were not directly admitted to an interventional center.^4 13^ As described by the INTERRSECT study and confirmed by our results, TM is mainly determined by thrombolysis and time.^3^ Since needle-to-groin times may be about twice as long in patients treated in a drip-and-ship manner,^19^ TM rates might be significantly higher in these patients, leading to increased rates of (partial) reperfusion before reaching an interventional center. Therefore, it is crucial to include these patients in further clinical trials investigating non-inferiority of MT only. Otherwise, transferability into real-life clinical practice is limited. To our knowledge, this is the first large cohort study distinguishing between ICA-T occlusions and ICA occlusions with patent circle of Willis. We found evidence that TM rates are considerably higher in patients with patent circle of Willis and that TM may lead to clinical deterioration in these patients. One small, monocentric study reported a worse clinical outcome in patients with TM from proximal LVO into the previously patent circle of Willis. However, this study combined pretreatment TM and TM as a complication during EVT, blurring its true impact.^8^ The phenomenon of TM from very proximal occlusions warrants further studying. In patients treated with IVT, we observed a higher prevalence of TM in individuals with distally located LVO (aOR 2.14 (1.10 to 4.15), P=0.03). These findings are concordant with the INTERRSECT study, and previous studies regarding reperfusion status after IVT alone or as a bridging therapy before EVT.^3 20 21^

Our data suggest that stroke etiology might be another relevant factor associated with TM. In patients with LVO stroke due to LAA, the prevalence of TM was halved, when compared with other stroke etiologies. Previous reports on a possible association between TM and stroke etiology do not give a clear picture yet. In line with our results, a large imaging study reported a higher rate of TM in cardioembolic thrombi when compared with other stroke etiologies including LAA.^5^ Conversely, the MR CLEAN Registry reported a higher prevalence of symptomatic cervical ICA obstruction or occlusion in TM patients.^4^ We propose two possible explanations for the lower rate of TM in patients with LAA stroke that we observed in our data. First, in patients with an intracranial stenosis, the stenosis itself might serve as a natural, mechanical barrier against downstream migration. Second, in LVO caused by a ruptured atherosclerotic plaque, clot composition may differ from cardioembolic thrombi, making the clot less susceptible to thrombolysis and subsequent TM. Given these findings, the impact of stroke etiology on TM remains controversial and further studies investigating this subject are justified.

### Clinical and technical outcomes

Most interestingly, TM impaired the chance to achieve complete reperfusion (as defined by mTICI 3), but TM was not associated with worse clinical outcome after 3 months. This finding corroborates two previous reports showing that TM does indeed worsen technical but not clinical outcome.^3 4^

### Limitations

Limitations of our study include the bicentric design making it prone to selection bias due to center-specific factors. Comparability withother reports may be hampered by definitions and graduations of TM used.^3–5^ Due to the relatively low prevalence of TM, we were not able to adjust for distance of TM (one or several segments downstream) in a meaningful way. Imaging assessment was not performed in a central imaging laboratory. However, the blinded assessment regarding thrombolysis status and other clinical variables still ensured high quality of data. The inclusion of different modalities of imaging (CTA/MRA) may have influenced precision when determining the exact location of target occlusion. Since we did not assess collateral status in the DSA (contrast fluid injection in the non-occluded ICA) before MT was performed, we were not able to detect pretreatment clot fragmentation (meaning persistence of the most proximal occlusion but evidence of downstream embolization of thrombus fragments).

## Conclusions

TM is a common phenomenon in LVO stroke that impedes complete recanalization but does not seem to worsen clinical outcome. TM is time dependent and has a strong association with thrombolysis. Given these findings, further investigations in patients with LVO stroke treated by a drip-and-ship manner are needed to assess the impact of TM in real-life clinical practice.

10.1136/jnis-2022-019365.supp2 Supplementary data

## Data Availability

Data are available upon reasonable request.
