# Supplementary material for: Thrombus migration in ischemic stroke due to large vessel occlusion: a question of time
Source: J Neurointerv Surg. 2022 Nov 1;15(e2):e216–22. doi: 10.1136/jnis-2022-019365 (PMC10646911; doi:10.1136/jnis-2022-019365)
Supplement: Supplementary data [file jnis-2022-019365supp001.pdf]

Thrombus migration in Ischemic Stroke due to Large Vessel Occlusion: A question of time

Supplement

Classification of occlusion site:

Localization of thrombus was classified as follows: Extracranial internal carotid artery (ICA extracranial), intracranial ICA with patent circle of Willis (ICA-I), carotid-T (ICA-T), proximal M1 segment of the middle cerebral artery (MCA M1 prox), distal MCA M1 segment (MCA M1 dist), proximal M2 segment of the MCA (MCA M2 prox), distal M2 segment of the MCA (MCA M2 dist), occlusions in the MCA M3/M4 territory and the anterior cerebral artery (ACA).

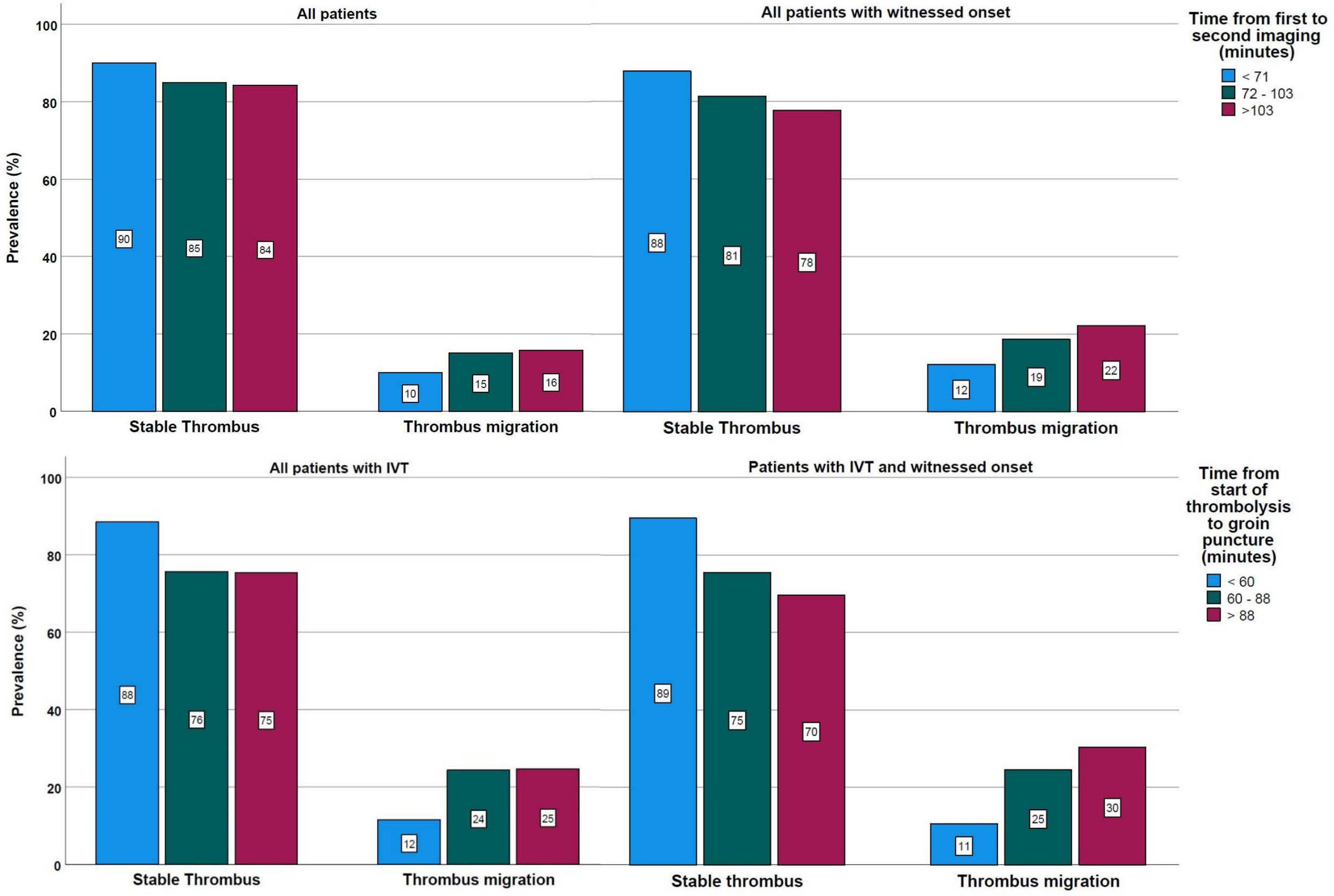

Figure S1: Time-dependency of Thrombus migration across subgroups, stratified by tertiles
